# Letter to the Editor regarding article “Association between electrocardiographic features and mortality in COVID‐19 patients”

**DOI:** 10.1111/anec.12869

**Published:** 2021-07-17

**Authors:** Elham Mahmoudi, Mohammad Keykhaei, Hirad Yarmohammadi, Reza Mollazadeh

**Affiliations:** ^1^ Universal Scientific Education and Research Network (USERN) Tehran University of Medical Sciences Tehran Iran; ^2^ Endocrinology and Metabolism Research Institute Non‐Communicable Diseases Research Center (NCDRC) Tehran University of Medical Sciences Tehran Iran; ^3^ Division of cardiology Columbia University New York NY USA; ^4^ Department of Cardiology School of Medicine Imam Khomeini Hospital Complex Tehran University of Medical Sciences Tehran Iran

**Keywords:** basic, electrophysiology ‐ cardiac arrest, clinical, clinical, ventricular tachycardia, fibrillation, non‐invasive techniques ‐ electrocardiography, sudden death

To the Editor,

With interest, we read the study recently published in Annals of Noninvasive Electrocardiology by Antwi‐Amoabeng et al., (2021) describing electrocardiographic predictors of mortality in coronavirus diseases 2019 (COVID‐19) patients. The authors also illustrated that the post‐infection corrected QT interval was similar to the baseline value.

Corrected QT interval (QTc), as a marker of ventricular repolarization duration, has been used to assess the risk of developing cardiac arrhythmias and sudden cardiac death (SCD) since 1952. It has been a major concern throughout the pandemic due to the concomitant use of COVID‐19 medications and reports of SCD in severe cases (Laleh Far et al., 2020; Shirazi et al., 2021). However, the current study has not reported the QTc interval values in different study groups and its correlation with QT‐prolonging agents. Besides, in the following sentences, we like to suggest another marker of SCD to be assessed in severe cases of COVID‐19 patients.

According to the predisposing role of regional repolarization heterogeneity in developing ventricular reentries, electrocardiographic indicators of repolarization disparity have been proposed to predict the risk of lethal cardiac arrhythmias.

QT interval dispersion (QTd) among 12 leads of standard surface ECG has been suggested to be a predictor of ventricular arrhythmias, as it described the lower risk of pro‐arrhythmia seen with amiodarone usage comparing to other anti‐arrhythmic agents. However, further studies rejected these findings and its predictive value is now under question.

The interval between peak of T‐wave (Tp) to end of T‐wave (Te) represents the termination of repolarization in epicardial and M cells, respectively, and it is a marker of transmural repolarization disparity (Figure 1). The main determinant of Tp‐Te interval is the action potential duration of M cells, carrying the longest action potential duration and being highly susceptible to delayed afterdepolarizations induced by drugs and intrinsic/extrinsic mediators. Therefore, the changes in Tp‐Te interval would assess cardiac electrical instability due to systemic inflammations and variable treatment protocols.

**FIGURE 1:**
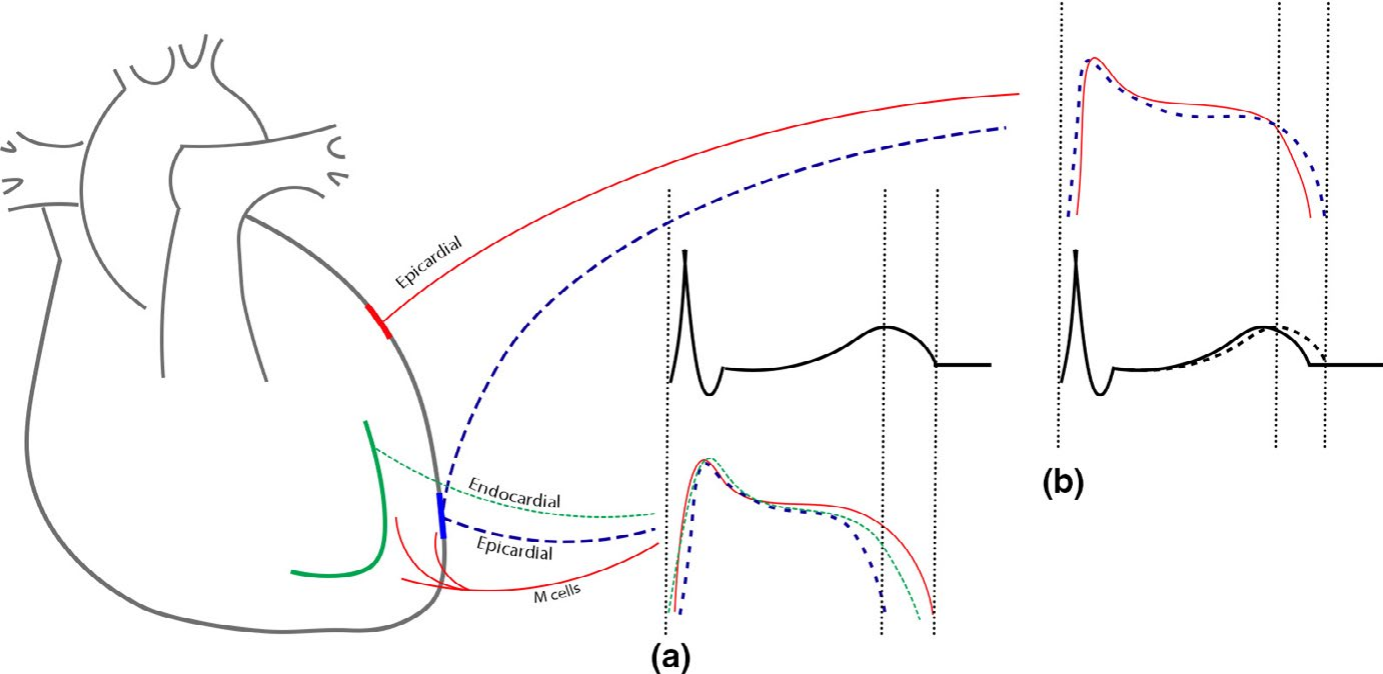
A schematic description of T‐peak to T‐end and QT dispersion: Indicators of transmural and regional disparity of repolarization, respectively. (a) The interval from the peak of T wave to the end of T wave represents the transmural temporal differences in repolarization termination between epicardial and M cells (b) The solid and the dashed lines simulate the repolarization duration of M cells recorded from two different regions of myocardium. Differences between the endings of the repolarization curves, concomitant with the end of T waves, represent on surface ECG as variable QT intervals

Tp‐Te interval has been reported to be prolonged in a group of 120 COVID‐19 patients, and Tp‐Te duration was correlated with the severity of the infection, though QTc was similar to healthy controls (Koc et al., 2020). Also, in another study by Shaghee et al. (2021), COVID‐19 pneumonia outcome was significantly correlated with both the QTc and Tp‐Te interval, though the latter marker had more prominent changes as the Tp‐Te to QTc ratio was also significantly increased in more severe cases.

Taken together, we suggest that Tp‐Te interval should be examined as an available robust method to predict the risk of cardiac arrhythmia in COVID‐19 patients, considering the existed limitations of performing cardiovascular imaging and bedside history taking in severe cases of COVID‐19.

Eventually, we should mention that the indexed article by Amoabeng et al. contains a figure to represent the prevalence of electrocardiographic features in COVID‐19 patients before and during the infection. Since the pre‐infection ECGs are available for only half of the patients, comparing the raw values of two groups is not reasonable and it would be more sensible to report the prevalence of ECG features per total records in each group.

## DISCLAIMER

The views expressed in this manuscript are our own, and they are not contributed to any institution or funder.

## CONFLICT OF INTEREST

There is no conflict of interest.

## AUTHOR CONTRIBUTIONS

Dr EM and MK wrote the primary draft. Dr RM and HY mde the revsions. EM made the initial idea.

## DATA AVAILABILITY STATEMENT

Data is avaliable on demand.
